# Personal and Psychological Traits Influencing the Willingness to Pay for Food with Nutritional Claims: A Comparison between Vice and Virtue Food Products

**DOI:** 10.3390/foods9060733

**Published:** 2020-06-03

**Authors:** Belinda López-Galán, Tiziana de-Magistris

**Affiliations:** 1 Unidad de Economía Agroalimentaria y de los Recursos Naturales, Centro de Investigación y Tecnología Agroalimentaria de Aragón, 50059 Zaragoza, Spain; belindasusanlopez@gmail.com; 2 Instituto Agroalimentario de Aragón (IA2), CITA-Universidad de Zaragoza, 50013 Zaragoza, Spain

**Keywords:** psychological traits, food choices, nutritional claims, emotional eating, STAI-T, body image, body mass index, WTP-space

## Abstract

Several studies have demonstrated the usefulness of nutritional claims (NCs) to help consumers make more informed food decisions. However, fewer studies have investigated the effect of personal and psychological consumer characteristics on their food purchase decision. Hence, the main aim of this study is to examine the impact of body image, emotional eating style, anxiety traits, and body mass index on consumer preferences for multiple NCs on the front of the package (FOP) of hedonic (potato chips) and utilitarian (toasted bread) products carrying the same NCs. Therefore, a real choice experiment (RCE) has been used and the willingness-to-pay-space model was estimated to analyse the interaction between personal and psychological characteristics and consumer preferences. The main finding suggests differences in purchase behaviour between potato chips and toasted bread. In particular, consumers are willing to pay more for toasted bread with one NC than potato chips with one NC. Moreover, personal and psychological consumer characteristics influence the purchase behaviour regarding food products with nutritional claims. However, only the anxiety trait appears to explain the differences between the selection of both types of food products. In line with this new evidence, we proposed some behavioural, political, and managerial implications.

## 1. Introduction

Non-communicable diseases (NCDs) are the primary cause of death in high-income countries [1], where empirical evidence confirms that these illnesses are related to high consumption of ultra-processed foods that are rich in low-quality fats, salt, and sugar [2,3]. Likewise, the increase of NCDs also has macroeconomic negative impacts on public health expenditure, reduction of life expectancy, and reduced productive capacities [4,5,6].

In this context, policymakers advise consumers to increase their consumption of fresh fruit and vegetables and to reduce their intake of sugar, saturated fats, and salt [7]. Likewise, several food-labelling systems have been introduced to help consumers make more informed food choices. An example is the introduction of the European Union (EU) regulation on nutritional claims (NCs) and health claims (HCs), which can be displayed on the front of the package (FOP) for food products (Regulation (EU) 1924/2006). While a nutritional claim (NC) is a statement that informs consumers of the quantity of one or more nutrients modified during the production process (e.g., reduction in fat, salt, sugar, etc.) and thus of their perceived benefits to human health, the health claims approved by European Food Safety Authority (EFSA) communicate clear and scientifically proven potential health benefits stemming from the consumption of products (e.g., reducing the consumption of saturated fat contributes to the maintenance of normal blood cholesterol levels).

During the last decade, extensive literature on NCs has been published. For example, studies have confirmed that the purchase of food products differs among utilitarian (i.e., healthy food) and hedonic food products (i.e., comfort food), mainly due to the different goal that these food products evoke to consumers [8,9,10,11] and different types of expectations. For example, the perception of utilitarian food as healthy, and carrying a nutritional claim, can be different from the perception of a hedonic food that is perceived as unhealthy, where consumers develop poorer taste expectations in response to seeing a nutrition claim on these items [10,11,12,13]. Some studies have reported that consumers’ purchase of hedonic foods endorsed with nutrition claims might be lower [14,15,16] because consumers are sceptical of the nutrition claims [17]. This evidence is confirmed by Machín et al. [18], who conducted a choice task and found that providing a health goal to consumers resulted in the largest positive effect on products with front-of-package (FOP) labels, particularly in those with a healthy image (e.g., yoghurt, cheese, etc.) compared to an unhealthy image (e.g., snack, etc.). More recently, Gracia and Barreiro-Hurlé [19] compared two types of cereal-based food products (less energy-dense biscuits and more energy-dense pastries). The authors reported that Spanish consumers ranked the importance of fibre and reduced-fat claims differently. For example, in the case of biscuits, consumers attached more importance to the source of fibre followed by the reduced-fat claims. However, for pastries, consumers attached more importance to reduced-fat claims followed by claims regarding the source of fibre. Similarly, Roseman et al. [20] revealed that American consumers increased their perception of healthiness if a healthy food product included FOP labels. However, an unhealthy food product with an FOP label was not perceived as healthier.

On the other hand, the differences in purchase behaviour between utilitarian and hedonic food products with NCs might also be influenced by personal and psychological factors. These factors have been extensively addressed in the literature on eating behaviour, such as body image [21,22,23], emotional eating style [24,25], and anxiety tendency [26,27], which could be strongly related to consumer shopping behaviour. To illustrate, some studies showed that certain individuals react to external stimuli by increasing their consumption of indulgent food products (i.e., energy-dense food product) as a coping strategy. This eating behaviour, called emotional eating, is due to their inability to manage their negative emotional states [24]. These individuals had a high risk of suffering from eating disorders [28] or difficulty maintaining their weight loss [29].

Likewise, another construct, known as body image, recognises the individual’s perceptions and attitudes towards how they see their own body and how they feel about what they see [30]. Empirical evidence suggests that those individuals who have body image dissatisfaction, meaning they have a negative subjective valuation about their own body, tend to follow an unhealthy diet and are adversely influenced by the consumption of high-calorie food and, in extreme cases, eating disorders [21,22,23].

Finally, anxiety is another personal trait that affects eating behaviour. The anxiety trait characterises some individuals who tend to perceive their context (e.g., situations, other people, etc.) as dangerous and threatening, which results in their feeling nervous. Hence, they somatise this psychological state in physical symptoms, such as hyperventilation or high blood pressure [31]. Some authors highlighted that individuals with a high score on anxiety tend to present a high consumption of comfort food as a coping strategy to deal with their negative emotional state [26,27].

While these studies are important and contribute to the literature of consumer eating behaviour, we believe that use of personal and psychological traits in purchase behaviour as drivers in the consumer’s decision to purchase utilitarian and/or hedonic food products have not been fully exploited [32,33,34,35,36], in particular with regards those bearing NCs. To our knowledge, only a few studies have recognised psychological traits. For example, Bazzani et al. [32], Lin et al. [35], and Grebitus et al. [34] investigated the interaction between personality characteristics known as the “Big Five personality traits” and consumer preferences for apple sauce, genetically modified pork product, and food mile label, respectively. However, de-Magistris et al. [33] and López-Galán and de-Magistris [36] used the body image state and emotional eating style to examine the relationship between these personality traits and preferences for food carrying NCs. Consequently, more research is needed to contribute to this scant literature.

Given the aforementioned framework, the objective of the research is to consider other relevant psychological traits, such as body image, emotional eating style, anxiety traits, and body mass index (BMI) to investigate their effect on consumer preferences for multiple NCs presented on FOP labels of a utilitarian food (i.e., toasted bread) and hedonic food (i.e., potato chips). To achieve this objective, a real choice experiment (RCE) was used.

This study is the first to analyse consumer preferences for multiple (more than one) NCs, considering the relationship between the psychological constructs and purchase behaviour. It, furthermore, compares two products from two different product categories and uses the same NCs as a baseline. Finally, the results of this study provide new evidence that allows stakeholders to improve the efficacy of public health policies.

## 2. Materials and Methods

### 2.1. Real Choice Experiment

The choice experiment (CE) is the most widely stated preference method to evaluate consumer demand for food products. This method is based on Lancaster’s consumer theory [37] and random utility theory [38], where respondents participate in multi-choice tasks consisting of choosing one alternative from several alternatives of a product, each with different attributes and levels. As in real purchase situations, they are required to choose what, if any, from the offered items they would purchase.

Nevertheless, hypothetical CE has been criticised because of the presence of hypothetical bias. In other words, participants overstate their willingness to pay (WTP) for the product when their choices have been made without any financial commitments [39,40]. To overcome this obstacle, this study conducted a real choice experiment (RCE). RCE is a non-hypothetical method that considers an incentive-compatible mechanism and real food products. Hence, the economic valuations made by consumers are consistent with their actual purchase behaviour.

### 2.2. Recruitment and RCE Procedure

We conducted the present study during March and April of 2015 in Zaragoza, Spain. This city was selected because the sociodemographic profile and level of income of the sample was representative of the Spanish population. Hence, the results of the present study can be extrapolated to the whole country [41].

The experiment followed the guidelines of two ethical protocols: The Declaration of Helsinki and the protocol approved by the Ethics Committee of Centro de Investigación y Tecnología Agroalimentaria de Aragón-CITA (FP7-MC-CIG-332769). To specify the sample size, we considered a sample error of ± 7% and a confidence level of 95.5% (K = 2) when estimating proportion (*p* = q = 0.5). Moreover, to ensure the optimal sample size, we carried out a power analysis following Cohen’s method [42]. The prior power calculation analysis resulted in a medium effect size of d = 0.80, with α = 0.05, and finally, the post-analysis resulted in a power of 0.995. Consequently, 310 individuals were recruited for the experiment by a subcontracted professional market research agency. We used a stratified sampling procedure according to gender, age, education level, and BMI. After dropping the data of five participants because they did not complete the entire questionnaires, the final sample resulted in 309 individual who evaluated the potato chips and 306 individuals who evaluated the toasted bread. Participants were consumers of toasted bread and/or potato chips older than 18 years and primary food buyers in their households.

Several sessions were performed for the experiment, where a maximum of 10–12 participants were invited in each session and they received the same treatment. To avoid any communications among the subjects, the sessions were conducted in a large room and each participant was seated separately and far from other participants. The experiment was conducted in the following steps. The first step consisted of asking each participant to read and sign an informed consent form. After assigning a unique ID number to each participant to guarantee his/her anonymity before beginning the experiment, participants were informed that they would receive 10 euro in cash at the end of the session for taking part in the experiment. The second step consisted of describing and explaining to participants each of the products in the choice sets. Thereafter, they were asked to inspect the different products in the choice sets because they would take part in two choice tasks (task I for the potato chips and task II for the toasted bread). Finally, participants were informed that at the end of the experiment, the experimenter would randomly select one of the tasks to be binding. Then, when one of two tasks had been randomly selected as binding, a number between 1 and 12 (the total number of choice sets) would randomly be selected to determine the binding choice set. The participants would then buy the product they had chosen in this binding choice set and pay the corresponding price unless they selected the no-buy option.

### 2.3. Product and CE Design

Food product categories considered for analysis were potato chips and toasted bread. Potato chip are the most consumed snack in Spain, albeit occasionally [43], according to Mercados Centrales de Abastecimiento, S.A.(MERCASA) [44]. However, toasted bread is one of the most consumed items in Spain (10%) out of all processed bread. Hence, by comparing a product with hedonic value (potato chips) and another with utilitarian value (toasted bread), we were able to identify differences in preferences for different products that carry the same nutritional claims.

To facilitate the comparison of the results between the two food products, the same attributes and levels were used in the RCE. Table 1 shows that the attributes selected were price and two nutritional claims. The first attribute (PRICE) is based on the prices found at the Spanish supermarket at the time of the experiment. To include an extended price range and to compare consumer willingness to pay for the nutritional claims, four levels were considered with an incremental of €0.45 between each level in both food categories. Price levels for potato chips were €0.50, €0.95, €1.40, and €1.85, and for toasted bread, they were €0.70, €1.15, €1.60, and €2.05. The second attribute (FAT) represented the reduced-fat claim, which indicated that the food product was produced with a 30% reduction in fat compared to the traditional version of the food product. The last attribute (SALT) represented the low-salt-content claim, which denoted that the products contained not more than 0.03 g of salt per 100 g. Finally, we also considered the interaction between both nutritional claims (FSALT) by the inclusion of reduced-fat and low-salt claims jointly on FOP.

The selection of these claims is in line with the findings regarding some nutrients that are directly related to obesity and other non-communicable diseases [3,7]. In particular, Hooper et al. [45] demonstrated that a low-fat intake helped people to reduce the risk of weight gain in normal-weight people or to reduce the weight of obese and overweight people. On the other hand, Fernández et al. [46] and the World Health Organization (WHO) [7] related the prevalence of non-communicable diseases (e.g., cardiovascular illnesses) to intakes rich in fat. Finally, Zhu et al. [47] found a positive relationship between high-salt intake and some obesity measures. In this context, the WHO [7] recommends reducing salt intake to 5 g per day because high consumption of this nutrient increases the risk of kidney disease, osteoporosis, and high blood pressure [48,49].

To reduce the hypothetical bias, the D-error was minimised by considering a sequential Bayesian in the experimental design. This approach is termed Efficient Design because it presents the lowest possible standard error by separately measuring the taste preference of each nutritional claim [50,51]. The choice design was determined with the Ngene software version 1.1.2 (ChoiceMetrics, Sydney, Australia) [52].

**Figure 1:**
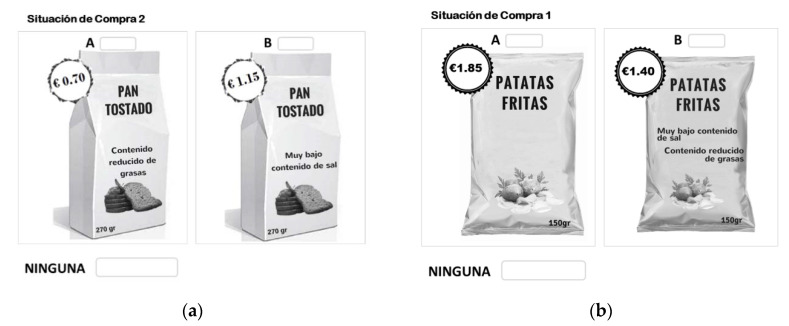
(**a**) Example of a choice set of toasted bread, and (**b**) example of a choice set of potato chips.

According to Scarpa et al. [51], we followed three steps to determine the choice design by performing the sequential Bayesian approach. In the pilot, we assumed multinomial probability specification to derive the design. Hence, we selected attributes and their level to derive an orthogonal factorial design. We then used the data from the pilot study to estimate a model whose coefficient estimates were then used as Bayesian priors. This last model determined an efficiency of 96.6% required by a choice design with 12 tasks. Moreover, each choice task must be structured with two different food product options (potato chips or toasted bread) and a no-buy option (Figure 1).

### 2.4. Measures

In this study, three scales and one bodyweight measure were used to identify the influence of personal and psychological factors on the purchase behaviour of food products that bear nutritional claims. Two are related to the influence of negative emotions (emotional eating scale, trait anxiety inventory subscale) on behaviour, while the other scale indicated the level of satisfaction that an individual has with her/his own body (body image scale), and the last one indicated the weight status of the participants (body mass index).

The emotional eater questionnaire (EEQ) [53] assesses the impact of negative emotions on the eating behaviour of an individual. This scale is structured around ten items rated on a four-point system (never, sometimes, generally, and always) (see Supplementary Materials Table S1 and Table S2). Lower scores indicate that individuals control what they eat since they decide to eat only when they are hungry. Higher scores indicate a higher tendency for negative emotional states of consumers to determine how and how much they eat. In this study, the Cronbach’s alpha calculated was 0.82 for potato chips and 0.86 for toasted bread, indicating very good reliability and an adequate measure of the EEQ in the samples.

Anxiety is an emotional state in which the individual suffers several intense emotions, such as apprehension, tension, and nervousness [54]. In addition to these emotions, the individual may experience many physiological alterations, such as raised blood pressure or hyperventilation [54]. A subscale measures anxiety (AX) as an individual characteristic [54,55]. This scale contained 20 items rated on a four-point system (rarely, sometimes, often, and almost always). A lower score indicates a lower level of anxiety. The Cronbach’s alpha was 0.90 for potato chips and 0.89 for toasted bread, which is an excellent indicator of the reliability and adequacy of the scale in the samples.

Body image scale (BISS) measures the favourable or unfavourable perception of an individual about his or her own body [30]. This scale contains six items that examine individual experiences regarding the following: (1) overall physical appearance, (2) body size and shape, (3) weight, (4) physical attractiveness/unattractiveness, (5) current feelings regarding one’s looks compared to how one typically feels, and (6) evaluation of one’s appearance compared to the average person’s appearance. The participants answered a 9-point bipolar Likert scale, which measured how the respondents felt about the six items at that moment in the experiment. This scale classified participants into two groups: people with a positive body image (self-accepting) and people with body image dissatisfaction. The Cronbach’s alpha of BISS was 0.74, which represents an acceptable reliability and adequacy index of the scale in both food products. Body mass index (BMI) is the weight status of an individual that is obtained from the ratio of weight in kilograms (kg) to the height in square metres (m^2^) [56]. The WHO determined that the risk of suffering non-communicable diseases was the same as the average person with a normal weight status (less than 24.9 kg/m^2^), it increased in people with pre-obesity or overweight status (between 25.0 and 29.9 kg/m^2^), but it was severe when an individual was obese (more than 30 kg/m^2^). Mixed evidence indicates a relationship between body mass index (BMI) and psychological characteristics. Some studies report a clear relationship between BMI, body image [57], anxiety [27], and emotional eating [58]. However, some studies did not determine any relationship between BMI and emotional eating style [59].

### 2.5. Model Specification

According to the economic theories of Lancaster [37] and McFadden [38], consumer utility is a random variable that can be obtained when an individual, *n,* faces a choice among j alternatives in one of t choice occasions. This utility can be represented as follows:

U_njt_ = V_njt_ + ε_njt_ (1)

In this assumption, the V_njt_ represents the utility broken down into the product attributes and the ε_njt_ is a random term that is independent and identically distributed (iid) over time, people, and alternatives.

Many models specify the utility, but the most appropriate models consider heterogeneity in consumer preferences [19]. In this regard, mixed logit models capture this heterogeneity. This group of models assumes that both the utility of component attributes and the error term differ across individuals. This approach of heterogeneity is known as scale heterogeneity [60]. However, following the methods of Scarpa, Thiene, and Train [61], we reparametrized the utility function through the specification and estimation of the price coefficient distribution and the corresponding vector of the consumer’s WTP for each nutritional claim [62]. Hence, the parameters’ coefficients are interpreted as marginal WTP values. Therefore, the utility function can be represented as follows:

U_njt_ = θ_n_ (−PRICE_njt_ + β_n1_FAT_njt_ + β_n2_SALT_njt_ + β_n3_FSALT_njt_ + NOBUY) + ε_njt_ (2)

where θ_n_ is the random positive scalar representing the price/scale parameter. PRICE_njt_ is a continuous variable that includes the four levels of price described in Table 1. FAT, SALT, and FSALT are dummy variables, where a value of 1 means that the corresponding claim is present on the package of the food product, and 0 indicates that it is absent, and NOBUY is an alternative-specific constant that represents a dummy variable of the no-buy option (i.e., NOBUY = 1, no-buy option; NOBUY = 0, otherwise). Moreover, β_s_ are the random coefficients of the estimated WTP values, and ε_njt_ is the error term that follows a Type I Extreme Value Distribution.

To analyse whether personal (BMI) and psychological characteristics (EEQ, AX, BISS) explain heterogeneity in space preferences regarding nutritional claims, we introduced the normalised means of the total score of each scale as a continuous variable in the model [32,35], and then we interacted it with the nutritional claims. This interaction can be represented in the following equation:

U_njt_ = θ_n_ (−PRICE_njt_ + β_n1_FAT_njt_ + β_n2_SALT_njt_ + β_n3_FSALT_njt_ + γ_1_EEQ_n_FAT_njt_ + γ_2_EEQ_n_SALT_njt_ + γ_3_EEQ_n_FSALT_njt_ + γ_4_AX_n_FAT_njt_ + γ_5_AX_n_SALT_njt_ + γ_6_AX_n_FSALT_njt_ + γ_7_BISS_n_FAT_njt_ + γ_8_BISS_n_SALT_njt_ + γ_9_BISS_n_FSALT_njt_ + γ_10_BMI_n_FAT_njt_ + γ_11_BMI_n_SALT_njt_ + γ_12_BMI_n_FSALT_njt_ + NOBUY) + ε_njt_ (3)

In Equation (3), we specified the scalar price/scale parameter (θ_n_), the PRICE variable, the NOBUY variable, and the variables FAT, SALT, and FSALT, as in Equation (2). Also, we introduced the γ_is_ parameter, which represents the coefficients of the interaction terms between each nutritional claim and each personal and psychological consumer characteristics. Because of this specification, we assumed that the coefficients were invariant within the sample and they measure the effect of the personal and psychological traits in willingness to pay for FAT, SALT, and FSALT claims for both food products. Thus, we estimated a mixed logit model specified in WTP space with correlated parameters.

To determine the robustness and consistency of the model, we considered changes in the Akaike information criterion (AIC) and log-likelihood (LL). We selected the model that has the lower values of AIC and LL. To estimate the final econometric model and to perform the statistical analysis, we used Nlogit software version 6 (Plainview, NY, USA).

## 3. Results

### 3.1. Descriptive Analysis of Sociodemographic and Psychologist Characteristics

Three hundred and nine individuals participated in the CE for potato chips, and three hundred and six participated in the CE for toasted bread. The sociodemographic characteristics of the sample and the population are presented in Table 2.

Potato chips and the toasted bread sample presented the same sociodemographic and psychological characteristics and neither demonstrated a significant statistical difference from the characteristics of the Spanish population. Therefore, the results of our study can be considered representative of the Spanish population since the final sample (hereinafter, when we refer to the sample, we refer to both potato chips and toasted bread samples) represents the population according to sociodemographic profiles. The majority of the participants were female (60%), which implies that women were overrepresented in comparison to the Spanish population. However, this difference is not statistically significant. The mean age was two years older than the mean age of the nation (43 years old) and 41% of the participants were in the middle age range, which is from 35 to 55 years old. Moreover, half of the sample have completed secondary studies (50%) and 38% had household incomes of between €1501 and €2500. On the other hand, almost half of the sample (49%) had normal weight, which is very similar to the proportion of people with normal weight in the country. Concerning the psychological characteristics, the sample presented a mean score of 32 points on the body image scale, which indicates that, in general, the sample has a positive opinion about their own body. Moreover, the sample presented a mean score of 12 on emotional eating, which suggests that, in general, the sample presents emotional eating behaviour that, while in this stage, certain negative emotions can influence the eating behaviour of the participants, most of the time they can control their food intake. The mean score of the anxiety tendency of the participants was 23 or, in other words, in general, this sample presented a medium tendency to anxiety, which is in line with 55% of the participants, which indicates a medium tendency to anxiety in both samples.

### 3.2. Personal and Psychological Factors Influencing Purchase Behaviour

Table 3 reports the estimated willingness-to-pay-space (€/package of product) parameters resulting from the mixed logit models in WTP-Space with correlated parameters. To determine whether the psychological characteristics of the sample influenced purchasing behaviour, we estimated first the basic WTP-Space model without the psychological interaction terms (model 1), and then we estimated the WTP-Space model including the psychological interaction terms (model 2) for each food product. As Table 3 reports, the inclusion of the psychological interaction terms in the econometric specification improves the performance of the model for both food products.

The log-likelihood (LL) decreased slightly from model 1 to 2 for potato chips and toasted bread, respectively. The same occurred for the Akaike Information Criterion (AIC). This small improvement in fit may indicate that there are other relevant factors (i.e., psychological and personal traits) that were not included in the model and should be considered in further studies. On the other hand, most of the interaction terms were statistically significant at 1%, except for BISS scale, which was not significant in both food products.

The negative and statistically significant coefficient in the no-buy option demonstrated that consumers preferred to buy any of the options of potato chips or toasted bread than choose the no-buy option. Furthermore, the participants presented an average WTP of €0.69 per package of potato chips with a reduced-fat nutritional claim in comparison with a conventional version of potato chips. Similarly, participants presented an average WTP of €0.39 per package of potato chips with a low-salt content claim. However, they showed an average discount of €0.40 per package of potato chips bearing both nutritional claims (reduced-fat and low-salt content). These WTPs were lower than those estimated for healthier toasted bread. More specifically, participants presented an average WTP of €1.03 per package of toasted bread with a reduced-fat nutritional claim and €0.61 per package of toasted bread with a low-salt content claim. However, participants appeared to require a discount of €0.41 to buy a package of toasted bread bearing both nutritional claims (i.e., reduced-fat and low-salt content). Finally, we observed that when both NCs were presented jointly on FOP, the WTPs for potato chips and toasted bread were similar. In general, these results indicate that consumers presented a higher average WTP for utilitarian products than hedonic ones carrying the same NCs.

As the results of model 2 indicate, the psychological traits affected preferences for nutritional claims. Regarding potato chips, the interaction term coefficients were statistically significant when the nutritional claims (FAT, SALT, and FSALT) interacted with the body mass index variable and the psychological scale, except the BISS scale. However, with regard to toasted bread, only the interaction of consumer body mass index and the emotional eating scale with all nutritional claims was statistically significant.

For potato chips with one nutritional claim (i.e., reduced-fat or low-salt nutritional claims), the emotional or very emotional eater participants presented a negative WTP. This result means that those participants who had less control over their eating behaviour would require a discount of €0.06 for potato chips with the FAT claim and €0.08 for potato chips with the SALT claim in comparison with those participants who had more control over their eating behaviour.

Moreover, participants with a high tendency to anxiety presented a price premium of €0.05 for potato chips with the FAT claim and showed a price premium of €0.06 for potato chips with the SALT claim. In relation to the BMI variable, the overweight and obese participants were willing to pay €0.07 more for a package of potato chips with FAT claims and presented the higher WTP for a package of potato chips with the SALT claim.

Finally, participants who were very emotional eaters had a higher evaluation of potato chips bearing both nutritional claims. However, those participants with a higher tendency to anxiety and higher body mass index required a large discount per package of potato chips bearing both reduced-fat and low-salt (FSALT) nutritional claims.

As for toasted bread with one nutritional claim, in general, the effect of the emotional eating pattern and the body mass index of consumers on purchasing behaviour was similar to potato chips, where their WTPs were higher. Specifically, the consumers who were emotional and very emotional eaters required the larger discount in the price to buy a package of toasted bread with a FAT claim (€0.09) or SALT claim (€0.12). On the other hand, those participants with higher BMI showed a positive WTP for toasted bread with a FAT claim (€0.07) and a SALT claim (€0.17).

However, participants who were emotional or very emotional eaters presented a higher valuation for toasted bread bearing both nutritional claims (FSALT) because their WTP was approximately €0.10 per package. Similarly, those participants with higher BMI would have required a discount of approximately €0.11 to buy a package of toasted bread when reduced-fat and low-salt claims appeared jointly on FOP.

## 4. Discussion

The results of our study are in accordance with López-Galán and de-Magistris [36] and Barreiro-Hurlé et al. [63], who reported that consumers showed a positive preference for products with one nutritional claim compared to those with two nutritional claims presented jointly on FOP. This result suggests that the presence of both NCs could present an information overload for the consumers, who did not evaluate their joint presence on FOP as a better alternative, preferring products with less complex nutritional information. However, our finding contrasts with findings in de-Magistris and López-Galán [64] and de-Magistris et al. [33], in which consumers reported positive preferences for cheese and potato chips bearing both reduced-fat and low-salt content claims, respectively. However, in line with Barreiro-Hurlé et al. [63], regardless of the type of food product included, more than one claim (FSALT) diminishes their utility. On the other hand, our results are dissimilar only in part with Ballco and Gracia [65], who reported that consumers were willing to pay premiums when NC fat-related HC in addition to the NC was presented, while not for the claims related to the fibre content.

Moreover, according to previous studies, the reduced-fat nutritional claim is the most preferred [19,33,36,64,66]. However, the WTP for the low-salt content claim is 44% and 41% less than WTP for reduced-fat claims in potato chips and toasted bread, respectively. This result is supported by Cavaliere et al. [66], who reported that only a quarter of their sample indicated an interest in this type of nutritional claims, and de-Magistris et al. [33], who found that 18% of their sample indicated a positive willingness to pay for low-salt content potato chips. In this regard, the taste can be an underlying factor that is more important than healthiness in the food purchase.

Another important finding of this study is the lower willingness to pay for hedonic potato chips with NCs than the WPs for utilitarian toasted bread. This result is following Machín et al. [18], Gracia and Barreiro-Hurlé [19], and Roseman et al. [20], but contrary to Barreiro-Hurlé et al. [63], Spanish consumers indicate a greater willingness to pay for toasted bread with one nutritional claim (FAT or SALT).

When we considered the impact of emotional eating, anxiety tendency, body image, and body mass index of consumers on the willingness to pay for nutritional claims, we surprisingly only found differences among products in anxiety tendency. In contrast with de-Magistris et al. [33], the body image of consumers appears to have no influence on purchase behaviour in any of the food products. This may be because this study considers more and other relevant psychological traits on consumer food choice.

On the other hand, in line with López-Galán and de-Magistris [36], emotional eating appears to influence purchasing behaviour in both food products similarly. More precisely, consumers that self-reported higher scores of emotional eating style indicate a minor willingness to pay for the reduced-fat and low-salt version of potato chips and toasted bread. Moreover, when we evaluated their willingness to pay for products bearing both nutritional claims, the consumers with a higher emotional eating style indicated the minor discount (i.e., the willingness to pay was negative) in the price for purchasing these types of products. However, this last finding contradicts the results of López-Galán and de-Magistris [36], who found that consumers with a higher emotional eating scale presented positive preferences for toasted bread bearing both nutritional claims, although this preference was not statistically significant.

Regarding the trait anxiety, we identified a clear influence on purchase behaviour. Specifically, we found that this trait was the only psychological trait that differed between the choice of potato chips and toasted bread. In particular, anxiety tendency was statistically significant only for potato chips. This finding is in line with eating behaviour literature in which higher scores of the anxiety trait were positively associated with hypercaloric food consumption [26,27]. However, when nutritional claims were considered as attributes in the food choice of this hedonic food product, we found a positive willingness to pay for reduced-fat, low-salt potato chips, and a negative willingness to pay for potato chips bearing both nutritional claims.

Whit respect to body mass index, this personal characteristic indicated that as consumers increased their body mass index, they increased their willingness to pay for reduced-fat and low-salt content potato chips and toasted bread. In the case of potato chips or toasted bread with two nutritional claims, they required a discount on the price of between €0.09 and €0.11 per package of reduced-fat and low-salt potato chips or toasted bread.

In the eating behaviour literature, among other factors, the higher body mass index appears to be related not only to the increased intake of energy-dense food products [67] but also unhealthy food choices [68]. Part of the reason that explains this pattern is a major tendency to anxiety and emotional eating style [26,27]. In this study, body mass index appears to assume a more relevant role in food purchase with nutritional claims than psychological traits. One possible explanation is that in our sample, the BMI and anxiety trait are uncorrelated (see Supplementary Materials Table S3 and Table S4). On the other hand, while the correlation between BMI and emotional eating style was statistically significant, the coefficient of correlation was less than 0.30, which means a small correlation between both factors [42]. This suggests that, in our sample, greater body mass index was not related to a lower ability to manage anxiety and negative emotions, but was due to other causes. However, this can be explained by the lower percentage of people with obesity (BMI greater than 30 kg/m^2^) in our sample.

Another interesting finding is that consumers with higher body mass index show a higher WTP for hedonic products with virtue characteristics. Consumers with higher BMI showed superior marginal WTP for potato chips with reduced-fat or low-salt claim (i.e., 10% and 38%, respectively) compared to toasted bread with the same NCs (i.e., 7% and 28%, respectively). This finding could suggest that the impulsivity and the self-control component (but to a lesser extent, emotional eating and anxiety also relate to these components) could underlie the food choices of these people. According to Chernev and Gal [69], consumers’ judgments appear to be against themselves, because they mistakenly think that the reduction of a nutrient means the reduction of the calorie content. Moreover, contrary to what we expected, these consumers presented higher WTP for a low-salt claim compared to a reduced-fat claim, which shows that, in these consumers, the taste is related to fat content and not to salt content. In this regard, the aforementioned bias in the judgment has a different effect according to the type of NCs.

Overall, this research revealed several interesting facts regarding consumer behaviour in response to food products with nutritional claims. First, the positive WTPs for hedonic products with one nutritional claim (FAT or SALT claim) confirmed the existence of the ‘health-halo’ effect [70]. In other words, the presence of a nutritional claim on an unhealthy food product (i.e., potato chips) can lead consumers to think that potato chips with a reduced-fat or low-salt claim are healthier food products because they assume that the positive valuation of the NCs is related to the product’s overall healthfulness. In this regard, contrary to its main purpose, NCs appear to act as a marketing strategy that provides more benefits to manufacturers and retailers than to the health of consumers. This argument is supported by Villas-Boas et al. [71], who showed that while product manufacturers increase their profits when they display only one nutritional claim in their reformulated food products, consumers do not always benefit.

Second, lower WTP for food products with a low-salt claim suggests that consumers assume that reduced salt content decreases the tastiness of the food products. This perception is more evident with the negative WTP for potato chips and toasted bread with two nutritional claims (FSALT claim). Third, we found differences in the purchase behaviour between hedonic and utilitarian food products. In particular, the WTP for a utilitarian food product (i.e., toasted bread) was higher, among 33% and 36%, for the reduced-fat and low-salt claim respectively, compared to the hedonic food product (i.e., potato chips). Fourth, our findings demonstrated that other and more relevant personal and psychological consumer characteristics influenced the purchase behaviour of food products with nutritional claims [33,36,72]. In particular, higher scores on emotional eating style affect negatively, while higher scores on anxiety trait and body mass index positively affect the WTP of food products with one nutritional claim. Conversely, higher scores on emotional eating style affect positively, while higher scores on the anxiety trait and body mass index influence negatively on WTP for food products with two nutritional claims. However, only the anxiety trait appears to explain differences in the purchase behaviour of Spanish consumers between hedonic and utilitarian food products.

This research faced some limitations that future studies can overcome. First, the econometric model used to analyse consumer preferences did not allow the identification of the direct or indirect effects of the personal and psychological factors on food purchase. Hence, future research can assess these preferences with hybrid models, such as Multiple Indicator Multiple Causes (MIMIC) models. Second, since this study explored typical hedonic and utilitarian food products, the perception of the tastiness of products with NCs can be related to the personal trait named “Unhealthy = Tasty Intuition” proposed by Raghunathan et al. [73], which links the expectations of taste generated by healthy food products. Unfortunately, this trait has not been considered in the study. Hence, future research could be taken into account to compare utilitarian and hedonic food products carrying NCs. Finally, because our sample presents a lower percentage of people with a high body mass index, future research should use a sample with a higher proportion of people with this characteristic.

From this study, we derived some behavioural, political, and managerial implications. First, because psychological factors appear to influence purchase behaviour, new approaches to decision-making process studies must be included in this perspective of the analyses. Second, it is not straightforward to provide solutions to the great issue of obesity and NCDs; however, a combination of strategies may facilitate the pathway. Some authors suggest that policymakers should introduce taxes or institute additional regulations for promoting unhealthy food products in mass media [74]. The present study highlighted that to facilitate more informed food choices, policymakers must provide instruments that provide consumers with an overall interpretation of the healthiness of a product, but by avoiding the health-halo effects that are characteristic of nutritional claims. According to some studies [75,76], other interpretative food labels such as health star rating or NutriScore are more efficient than nutritional claims. Moreover, policymakers should implement prevention strategies in the early stages of life by promoting educational campaigns that not only form in healthy dietary habits but to develop the correct coping strategies to confront negative emotional states. In adulthood, policymakers can use nutritional campaigns that promote mindful eating techniques on eating and purchasing behaviour or marketing strategies that encourage healthier eating habits using their consumer loyalty programs as instruments. On the other hand, since taste has been demonstrated to be relevant when consumers prefer to buy products reduced in salt, the food industry needs to invest more in research and development R+D in technological innovation to also produce healthy foods that are tastier than that of the competition. This strategy could reduce the negative halo-effect of healthy products carrying the NCs and, thus, increases their sales. Likewise, retailers should use a combination of marketing strategies [77] that help consumers to purchase not only healthier foods but also with the calorie content of their food intake, in accordance with energy content requirements.

## Figures and Tables

**Table 1:** Attributes and levels used in the choice experiment (CE) design for potato chips and toasted bread.

Attributes	Levels	
	Potato Chips	Toasted Bread
PRICE	€0.50	€0.70
	€0.95	€1.15
	€1.40	€1.60
	€1.85	€2.05
Reduce-fat claim (FAT)	0 = No label	
	1 = A reduced food product is reduced by at least 30% compared to the traditional version of the product.	
Low-salt content (SALT)	0 = No label	
	1 = The amount of salt in the food product is not more than 0.03 g of salt per 100 g of the product.	

**Table 2:** Sample sociodemographic and psychological characteristics.

Definition Variable	Population	Sample Potato Chips (*n* = 309)	Sample Toasted Bread (*n* = 306)
Sex			
Male	42.7	40.1	40.5
Female	50.9	59.9	59.6
Age (mean)	42.9	45.2	45.4
		(16.6) ^sd^	(16.6) ^sd^
From 18 to 34 years	24.1	28.5	27.8
From 35 to 55years	39.2	40.8	40.9
More than 55 years	36.7	30.7	31.4
Education Level			
Primary studies	17.0	19.7	19.9
Secondary studies	50.0	42.7	43.5
University studies	33.0	37.5	36.6
Household Income			
Below €1500	N/A	31.8	32.2
Between €1501 and €2500	N/A	38.3	38.8
More than €2500	N/A	29.9	29.0
Body Mass Index (mean) (BMI)		26.0	26.0
		(4.9) ^sd^	(4.8) ^sd^
Normal Weight (Below 25 kg/m^2^)	47.4	48.5	47.7
Overweight (Between 25 and 29.99 kg/m^2^)	35.7	32.7	33.0
Obesity (More than 30 kg/m^2^)	16.9	18.8	19.3
Body Image Scale (mean) (BISS)		32.3	32.2
		(6.6) ^sd^	(6.6) ^sd^
Good Body Image (BISS ≥ 30) BISS_H	N/A	68.9	69.9
Body Image Dissatisfaction (BISS < 30) BISS_L	N/A	31.1	30.1
Emotional eating questionnaire (mean) EEQ		12.2	12.2
		(6.1) ^sd^	(6.0) ^sd^
Non-emotional eater (score between 0 and 5)	N/A	15.5	15.0
Low emotional eater (score between 6 and 10)	N/A	26.9	27.1
Emotional eater (score between 11 and 20)	N/A	49.8	50.0
Very emotional eater (score between 21 and 30)	N/A	7.8	7.8
Trait Anxiety Inventory Subscale (mean) AX		23.2	23.2
		(9.6) ^sd^	(9.5) ^sd^
Low tendency to Anxiety (score between 0 and 20)	N/A	39.8	38.6
Medium tendency to Anxiety (score between 21 and 40)	N/A	55.3	56.9
High tendency to Anxiety (score between 41 and 60)	N/A	4.9	4.6

N/A, Not Available; kg/m^2^, kilograms/square metres; sd, standard deviation.

**Table 3:** Estimates from the Mixed Logit Models in willingness-to-pay (WTP)-Space (€/package of product).

	Model 1 (Without Interaction Terms)		Model 2 (With Interaction Terms)	
	Potato Chips	Toasted Bread	Potato Chips	Toasted Bread
Mean				
FAT	0.63	3.41	0.69	1.03
	(0.10) ***	(0.32) ***	(0.10) ***	(0.14) ***
SALT	0.51	1.66	0.39	0.61
	(0.09) ***	(0.24) ***	(0.10) ***	(0.13) ***
FSALT	−0.26	−0.66	−0.40	−0.41
	(0.09) ***	(0.21) ***	(0.10) ***	(0.13) ***
NOBUY	−1.47	−0.46	−0.94	−1.44
	(0.10) ***	(0.2877)	(0.09) ***	(0.14) ***
**Interaction terms with EE, AX, BISS, BMI**				
FAT × EE			−0.06	−0.09
			(0.02) ***	(0.02) ***
FAT × AX			0.05	0.01
			(0.01) ***	(0.01)
FAT × BISS			0.02	0.01
			(0.02)	(0.02)
FAT × BMI			0.07	0.07
			(0.02) ***	(0.02) ***
SALT × EE			−0.08	−0.12
			(0.02) ***	(0.03) ***
SALT × AX			0.06	0.01
			(0.01) ***	(0.02)
SALT × BISS			0.03	0.03
			(0.03)	(0.03)
SALT × BMI			0.15	0.17
			(0.03) ***	(0.03) ***
FSALT × EE			0.06	0.10
			(0.03) **	(0.03) ***
FSALT × AX			−0.05	0.01
			(0.02) ***	(0.02)
FSALT × BISS			−0.01	0.00
			(0.03)	(0.03)
FSALT × BMI			−0.09	−0.11
			(0.03) ***	(0.03) ***
**Standard deviation**				
FAT	1.11	4.62	1.11	1.75
	(0.09) ***	(0.34) ***	(0.08) ***	(0.17) ***
SALT	1.16	3.20	1.37	1.81
	(0.09) ***	(0.18) ***	(0.09) ***	(0.17) ***
FSALT	0.61	1.13	0.32	0.43
	(0.10) **	(0.17) ***	(0.10) ***	(0.14) ***
Log Likelihood	−2330.58	−2357.18	−2309.12	−2196.88
AIC	4691.2	4744.4	4672.2	4447.8
AIC/N	1.27	1.29	1.26	1.21

***, **, Indicate statistical significance at the 1% and 5% level. Number in parenthesis is standard error. FAT, reduce-fat claim; SALT, low-salt claim; FSATL, interaction between reduce-fat content and low-salt content claims; AIC/N, Akaike information criterion/size of sample; EE, emotional eating; AX, anxiety trait; BISS, body image scale; BMI, body mass index.

## Supplementary Materials

The following are available online at https://www.mdpi.com/2304-8158/9/6/733/s1: Table S1: Emotional Eater Questionnaire (EEQ) Garaulet (English Version). Table S2: Cuestionario del Comedor Emocional Garaulet (Spanish Version). Table S3: Correlation between psychologies constructs on potatoes chip. Table S4: Correlation between psychologies constructs on toast chip.
