# Supplementary material for: Personal and Psychological Traits Influencing the Willingness to Pay for Food with Nutritional Claims: A Comparison between Vice and Virtue Food Products
Source: Foods. 2020 Jun 3;9(6):733. doi: 10.3390/foods9060733 (PMC7353545; doi:10.3390/foods9060733)
Supplement: Supplementary file 1 [file foods-09-00733-s001.docx]

Supplementary Materials

**Table S1.** Emotional Eater Questionnaire (EEQ) Garaulet (English Version)

| Do the weight scales have a great power over you? Can they change your mood? | | | |
| --- | --- | --- | --- |
|   Never |   Sometimes |   Generally |   Always |
| Do you crave specific foods? | | | |
|   Never |   Sometimes |   Generally |   Always |
| Is it difficult for you to stop eating sweet things, especially chocolate? | | | |
|   Never |   Sometimes |   Generally |   Always |
| Do you have problems controlling the amount of certain types of food you eat? | | | |
|   Never |   Sometimes |   Generally |   Always |
| Do you eat when you are stressed, angry or bored? | | | |
|   Never |   Sometimes |   Generally |   Always |
| Do you eat more of your favorite food and with less control when are alone? | | | |
|   Never |   Sometimes |   Generally |   Always |
| Do you feel guilty when eat “forbidden” foods, like sweet or snacks? | | | |
|   Never |   Sometimes |   Generally |   Always |
| Do you feel less control over your diet when you are tired after work at night? | | | |
|   Never |   Sometimes |   Generally |   Always |
| When you overeat while on a diet, do you give up and start eating without control, particularly food that you think is fattening? | | | |
|   Never |   Sometimes |   Generally |   Always |
| How often do you fell that food controls you, rather than you controlling food? | | | |
|   Never |   Sometimes |   Generally |   Always |

**Table S2.** Cuestionario del Comedor Emocional Garaulet (Spanish Version)

| ¿La báscula, tiene un gran poder sobre ti? ¿Es capaz de cambiar tu estado de humor? | | | |
| --- | --- | --- | --- |
|  |  |  |  |
| Nunca | A veces | Generalmente | Siempre |
| ¿Tienes antojos por ciertos alimentos específicos? | | | |
|  |  |  |  |
| Nunca | A veces | Generalmente | Siempre |
| ¿Te cuesta parar de comer alimentos dulces, especialmente chocolate? | | | |
|  |  |  |  |
| Nunca | A veces | Generalmente | Siempre |
| **¿**Tienes problemas para controlar las cantidades de ciertos alimentos? | | | |
|  |  |  |  |
| Nunca | A veces | Generalmente | Siempre |
| ¿Comes cuando estás estresado, enfadado o aburrido? | | | |
|  |  |  |  |
| Nunca | A veces | Generalmente | Siempre |
| ¿Comes más de tus alimentos favoritos, y con más descontrol, cuando estás solo? | | | |
|  |  |  |  |
| Nunca | A veces | Generalmente | Siempre |
| ¿Te sientes culpable cuando tomas alimentos “prohibidos”, es decir, aquellos que crees que no deberías, como los dulces o snacks? | | | |
|  |  |  |  |
| Nunca | A veces | Generalmente | Siempre |
| Por la noche, cuando llegas a casa cansado de trabajar ¿es cuando menos control tienes con tu alimentación? | | | |
|  |  |  |  |
| Nunca | A veces | Generalmente | Siempre |
| Estás a dieta, y por alguna razón comes más de la cuenta, entonces piensas que no vale la pena y ¿comes de forma descontrolada aquellos alimentos que piensas que más te van a engordar? | | | |
|  |  |  |  |
| Nunca | A veces | Generalmente | Siempre |
| ¿Cuántas veces sientes que la comida te controla a ti en vez de tú a ella? | | | |
|  |  |  |  |
| Nunca | A veces | Generalmente | Siempre |

**Table S3.** Correlation between psychologies constructs on potatoes chip.

|  | EE | AX | BISS | BMI |
| --- | --- | --- | --- | --- |
| EE | 1.0000 |  |  |  |
| AX | 0.2701* | 1.0000 |  |  |
| BISS | -0.3144* | -0.4072* | 1.0000 |  |
| BMI | 0.2329* | n.s. | -0.4062* | 1.0000 |

***, **, * indicate statistical significance at 1%, 5% and 10% level

**Table S4.** Correlation between psychologies constructs on toast chip.

|  | CCE | AES | BISS | BMI |
| --- | --- | --- | --- | --- |
| EE | 1.0000 |  |  |  |
| AX | 0.2623* | 1.0000 |  |  |
| BISS | -0.3221* | -0.4051* | 1.0000 |  |
| BMI | 0.2462* | n.s. | -0.3776* | 1.0000 |

***, **, * indicate statistical significance at 1%, 5% and 10% level
